# Optimal flexion angle for graft fixation in lateral extra‐articular tenodesis combined with anterior cruciate ligament reconstruction: A systematic review

**DOI:** 10.1002/jeo2.70312

**Published:** 2025-07-02

**Authors:** Riccardo Compagnoni, Antonio Klasan, Alberto Grassi, Francesco Puglia, Stefano Zaffagnini, Pietro Simone Randelli, Jacques Menetrey

**Affiliations:** ^1^ Clinica Ortopedica, ASST Centro Specialistico Ortopedico Traumatologico Gaetano Pini‐CTO Milan Italy; ^2^ Department of Biomedical, Surgical and Dental Sciences Università degli Studi di Milano Milan Italy; ^3^ AUVA UKH Steiermark Graz Austria Graz Austria; ^4^ Johannes Kepler University Linz Linz Austria Linz Austria; ^5^ IIa Clinica Ortopedica e Traumatologica, IRCCS Istituto Ortopedico Rizzoli Bologna Italy; ^6^ U.O.C. Ortopedia e Traumatologia Pediatrica, ASST Gaetano Pini/CTO Milan Italy; ^7^ Laboratory of Applied Biomechanics, Department of Biomedical Sciences for Health Università degli Studi di Milano Milan Italy; ^8^ Swiss Olympic Medical Center, Hirslanden Clinique La Colline Geneva Switzerland; ^9^ Orthopaedic Surgery Service University Hospital of Geneva Geneva Switzerland

**Keywords:** ACLR, angle, graft, lateral extra‐articular tenodesis, Lemaire

## Abstract

**Purpose:**

This systematic review aims to investigate the knee flexion angles used for graft fixation in lateral extra‐articular tenodesis (LET) during anterior cruciate ligament reconstruction (ACLR) and their impact on clinical outcomes.

**Methods:**

Following PRISMA guidelines, MEDLINE/PubMed and EMBASE were searched up to February 2024. The inclusion criteria were original clinical studies (levels I–IV evidence) with at least 12 months of follow‐up, reporting knee flexion angles during LET graft fixation in ACLR. Exclusion criteria included non‐English articles, reviews, biomechanical studies, and case reports. Data on study design, patient numbers, fixation angles, and clinical outcomes were extracted, and study quality was assessed using the RoB 2 tool.

**Results:**

Out of 1134 studies identified, 21 met the inclusion criteria. The reported flexion angles for LET graft fixation ranged from full extension to 90°. Ten studies fixed the graft at 30°, consistently showing improved knee stability, reduced pivot‐shift rates, and better functional outcomes, especially in high‐risk patients. Three studies focused on graft fixation at 45° of flexion. Another four studies investigated fixation at 60° of knee flexion. Lastly, two studies assessed outcomes with fixation at 90°.

**Conclusion:**

LET combined with ACLR effectively restores knee stability across various flexion angles. While fixation at 30° is most commonly associated with positive outcomes, the lack of consensus on an optimal angle reflects differences in surgical techniques and patient‐specific factors. Further prospective research with long‐term follow‐up is needed to validate these findings and guide clinical practice toward optimal knee flexion angles for graft fixation in LET procedures.

**Level of Evidence:**

Level III.

**Abbreviations**
ACL: anterior cruciate ligament
ACLR: anterior cruciate ligament reconstruction
ACL‐RSI: anterior cruciate ligament‐Return to sport after injury scale
ALL: anterolateral ligament
ALLR: anterolateral ligament reconstruction
BTB: bone‐patellar tendon‐bone
DB ACLR: double‐bundle anterior cruciate ligament reconstruction
EMBASE: excerpta medica database
IKDC: international knee documentation committee
IT: iliotibial
LEAT: lateral extra‐articular tenodesis
LET: lateral extra‐articular tenodesis
LTF: lateral tibiofemoral
MEDLINE: medical literature analysis and retrieval system online
MRI: magnetic resonance imaging
PRISMA: preferred reporting items for systematic review and meta‐analysis
RCTs: randomised controlled trials
RoB: risk of bias
SB ACLR + LET: single‐bundle anterior cruciate ligament reconstruction combined with lateral extra‐articular tenodesis

## INTRODUCTION

The lateral extra‐articular tenodesis (LET) procedures, particularly the Lemaire procedure and its modifications, have shown significant efficacy in improving knee stability, in patients with anterior cruciate ligament (ACL) deficiencies [49]. This technique is widely accepted in the orthopaedic community and is often used alongside ACL reconstruction to effectively manage anterolateral rotational instability of the knee [37]. It is especially beneficial for patients who are at a higher risk of rotational instability or re‐injury, such as athletes or individuals with a history of knee injuries [23]. By reinforcing the lateral aspect of the knee and limiting internal rotation, the Lemaire procedure aims to reduce the strain on the ACL graft, thereby enhancing the overall stability of the knee joint and improving the long‐term success of ACL reconstruction surgeries [17, 27, 29].

Recent studies [1, 13, 20, 44] have explored various aspects of this procedure, including its impact on postoperative recovery, muscle strength, and the potential risks and benefits when combined with different ACL reconstruction techniques. The evolution of the Lemaire procedure reflects ongoing advancements in orthopaedic surgery and represents a crucial option in the surgical management of knee injuries, particularly in complex cases where additional support and stability are required [9, 31].

The degree of knee flexion during the fixation of the Lemaire procedure could be a critical biomechanical factor that influences the overall success of the intervention [7]. However, a definitive consensus regarding the optimal flexion angle for fixation remains elusive in the current literature. This systematic review aims to analyse the various flexion angles employed in lateral extra‐articular tenodesis (LET) procedures when combined with anterior cruciate ligament (ACL) reconstruction, as reported in clinical studies. The impact of different fixation angles on biomechanical stability and clinical outcomes associated with this combined surgical approach is being elucidated through the systematic evaluation of relevant parameters.

## MATERIALS AND METHODS

### Literature review

The authors conducted a systematic literature review following the PRISMA (Preferred Reporting Items for Systematic Review and Meta‐Analysis) guidelines for scoping reviews. The objective was to examine the influence of knee flexion degree during graft fixation in the Lemaire procedure. A comprehensive search was performed using MEDLINE/PubMed and Excerpta Medica/EMBASE databases to identify relevant studies. The search terms included: (“Lemaire procedure” [MeSH Terms] OR “Lemaire procedure” [All Fields]) AND (“Lateral extra‐articular tenodesis” [MeSH Terms] OR “Lateral extra‐articular tenodesis” [All Fields]). No date restrictions were applied, and articles published until 1 February 2024, were considered for inclusion.

### Eligibility criteria

The inclusion criteria encompassed original scientific articles with a level of evidence ranging from I to IV. Specifically, the review considered randomised controlled trials (RCTs), non‐randomised comparative studies, and prospective or retrospective cohort studies. Studies were included only if they detailed the degree of knee flexion for graft fixation in the LET technique when performed in conjunction with ACL reconstruction or revision. Additionally, only studies reporting clinical outcomes with a minimum follow‐up of 12 months were eligible.

Exclusion criteria included non‐English articles, studies that focused solely on surgical techniques without reporting clinical outcomes, expert opinions, letters to the editor, commentaries, conference abstracts, case reports, review articles, and biomechanical studies. Furthermore, studies involving additional surgical procedures beyond LET and ACL reconstruction were excluded from the analysis.

### Study selection

**Figure 1:**
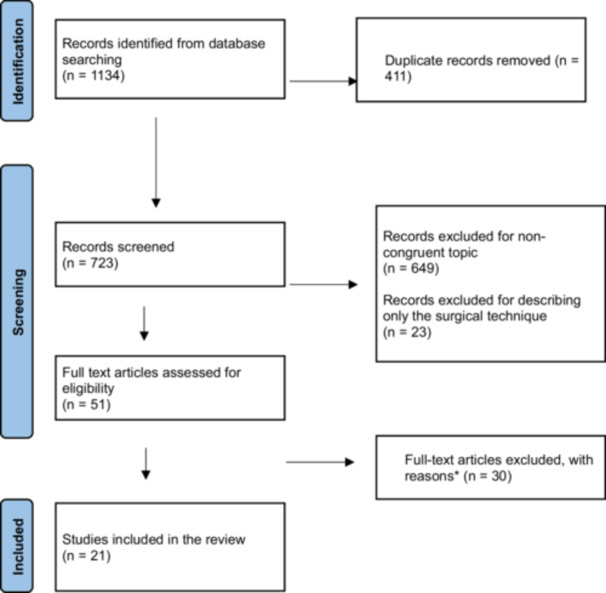
Selection criteria flow chart. *Not specifically examine only the lateral extra‐articular tenodesis (LET) combined with anterior cruciate ligament reconstruction (ACLR) or revision; cadaveric study.

Two independent reviewers (R.C. and A.K.) screened the identified studies based on their titles and abstracts, applying the predefined inclusion and exclusion criteria. In cases of disagreement, a third reviewer (A.G.) was consulted to reach a consensus. Following this initial screening, a full‐text review of the selected articles was performed independently by the two reviewers to finalise the inclusion of studies (Figure 1).

### Quality of studies assessment

The quality of the included studies was assessed using the Risk of Bias (RoB 2) tool (Table 1). Data extraction was conducted systematically by two authors, who compiled relevant information into a customised spreadsheet. The extracted data included study type, patient sample size, and the degree of knee flexion at the time of graft fixation in the LET procedure. Functional outcomes were assessed using subjective clinical scores (such as the Lysholm score, Tegner activity scale, and the subjective International Knee Documentation Committee [IKDC] scale) and objective clinical tests (such as the Lachman test, pivot‐shift test and objective IKDC classification).

**Table 1:** Risk of bias summary.

Study	Selection bias	Allocation concealment	Blinding of participants/personnel	Blinding of outcome assessment	Incomplete outcome data	Selective reporting	Overall risk
Ahn et al. [1]	Moderate	Not applicable	Unclear	Low	Some concerns	Low	Moderate
Alm et al. [2]	Moderate	Not applicable	Unclear	Low	Some concerns	Low	Moderate
Behrendt et al. [3]	Moderate	Not applicable	Unclear	Low	Some concerns	Low	Moderate
Branch et al. [4]	Moderate	Not applicable	Unclear	Low	Low	Some concerns	Moderate
Castoldi et al. [5]	Low	Low	Low	Low	Low	Low	Low
Chiba et al. [6]	Low	Low	Low	Low	Low	Low	Low
Declercq et al. [8]	Moderate	Not applicable	Unclear	Low	Low	Low	Moderate
Delaloye et al. [9]	Low	Low	Low	Low	Low	Low	Low
Deviandri et al. [10]	Moderate	Not applicable	Unclear	Low	Low	Some concerns	Moderate
Di Benedetto et al. [11]	Some concerns	Unclear	No blinding	Some concerns	Some concerns	Low	Some concerns
Eggeling et al. [12]	No randomisation	No information	No blinding	Some concerns	Some concerns	Some concerns	High
Foissey et al. [13]	No randomisation	No information	No blinding	Low	Some concerns	Low	High
Frigout et al. [15]	High	Unclear	High	Some concerns	Unclear	Some concerns	High
Geeslin [16]	Low	Low	Low	Low	Low	Low	Low
Getgood et al. [18]	Low	Low	Some concerns	Low	Low	Low	Low
Gibbs et al. [19]	Low	Low	Some concerns	Some concerns	Low	Low	Low
Green et al. [20]	No randomisation	No information	No blinding	Low	Some concerns	Low	Some concerns
Hantouly et al. [21]	Some concerns	Some concerns	Some concerns	Some concerns	Low	Low	Some concerns
Herbst et al. [22]	No randomisation	No information	No blinding	Some concerns	Low	Some concerns	Some concerns
Inderhaug et al. [25]	Low	Not applicable	Not applicable	Low	Low	Low	Low
Jette et al. [28]	Low	Not applicable	Not applicable	Low	Low	Low	Low
Joseph et al. [29]	Moderate	Unclear	Unclear	Unclear	Low	Low	Moderate
Lagae [32]	Low	Not applicable	Not applicable	Low	Low	Low	Low
Marom [35]	Low	Not applicable	Not applicable	Low	Low	Low	Low
Marom [34]	Low	Not applicable	Not applicable	Low	Low	Low	Low
Mayr et al. [36]	Low	Not applicable	Not applicable	Low	Low	Low	Low
Neri [38]	Low	Not applicable	Not applicable	Low	Low	Low	Low
Novaretti et al. [39]	Low	Not applicable	Not applicable	Low	Low	Low	Low
Ozbek et al. [40]	Low	Not applicable	Not applicable	Low	Low	Low	Low
Pearce et al. [41]	Low	Not applicable	Not applicable	Low	Low	Low	Low
Perelli et al. [42]	Some concerns	Some concerns	Some concerns	Some concerns	Low	Low risk	Low
Rojas et al. [43]	Some concerns	Unclear	No blinding	Some concerns	Some concerns	Some concerns	Some concerns
Rowan et al. [44]	Some concerns	Some concerns	No blinding	Low	Low	Low	Low
Scheean et al. [46]	Low	Low	Some concerns	Low	Low	Low	Low
Shimakawa et al. [47]	Some concerns	Unclear	No blinding	Some concerns	Low	Low	Low
Sigloch et al. [48]	Some concerns	Unclear	No blinding	Low	Some concerns	Low	Some concerns

### Statistical analysis

Descriptive statistics were utilised to summarise the extracted data, reporting outcomes based on knee flexion angles at the time of graft fixation in LET combined with ACLR. Given the heterogeneity of included studies, a non‐pooled approach was adopted, presenting results per fixation angle.

Frequency distributions were used to compare the proportion of studies reporting specific fixation angles and their corresponding clinical outcomes.

Risk of bias was assessed using the RoB 2 tool, and studies were stratified based on their methodological quality. Where applicable, interquartile ranges (IQRs) were used to account for variations in outcome measures across different study designs. A qualitative synthesis was performed to evaluate trends and patterns in functional outcomes relative to the knee flexion angle at fixation, facilitating a structured interpretation of the data.

Due to the variability in study designs and the absence of uniform outcome reporting across studies, a formal meta‐analysis was not conducted.

## RESULTS

A total of 1134 articles were initially identified for this study. After the removal of duplicates, 723 articles remained for screening. Following the screening process and the application of inclusion and exclusion criteria, 21 articles were selected (Tables 2 and 3).

**Table 2:** Results of the literature search.

Authors	Date of pub.	N. of patients	Degree of flexion	Tibial rotation	Methods	Considerations
Frigout et al. [15]	2023	80	20°	Neutral	43 patients isolated ACLR vs. 37 patients ACLR + LET	LET does not significantly impact sagittal knee stability in the context of ACL reconstruction
Ahn et al. [1]	2020	95	30°	Neutral	ACLR single bundle + LET vs. double boundle	SB + LET better results in pivot‐shift and superior IKDC objective grades
Branch et al. [4]	2015	18	30°	Neutral	12 patients BTB reconstructions vs. 6 BTB + LET	Robotic testing indicated a significant reduction in maximum external rotation and a trend towards reduced total axial rotation in the ACL reconstruction with LET group
Castoldi et al. [5]	2020	120	30°	Neutral	61 knees isolated BTB ACLR vs. 60 knees BTB ACLR + LET	No significant differences in long‐term patient‐reported outcomes between the groups. trend toward decreased graft failure risk with the addition of LET
Declercq et al. [8]	2021	86	30°	Neutral	42 knees ACLR + Lemaire vs. 44 knees ACLR + Coker–Arnold	No significant differences in osteoarthritis development or clinical outcomes between the two techniques
Deviandri et al. [10]	2021	4	30°	Neutral	Fourpatients presenting with residual rotatory instability after ACLR	isolated LET could be an effective treatment option for patients experiencing residual instability post‐ACLR
Green et al. [20]	2023	49	30°	Neutral	Effectiveness of LET + ACLR with quadriceps tendon autograft in high‐risk adolescent patients.	favourable clinical outcomes over a 2‐year follow‐up, including a 0% graft rupture rate and a 100% return‐to‐sports rate
Hantouly et al. [21]	2023	100	30°	Not specified	Compared ACLR alone vs. ACLR + LET vs. ACLR + ALLR	No significant differences in terms of subjective stability, IKDC scores, ACL‐RSI scores, pivot shift grade, or subjective pain on activity
Perelli et al. [42]	2022	66	30°	Neutral	Effects of adding LET to ACLR in paediatric patients	Combining LET + ACLR reduced the cumulative failure rate and improved objective stability without increasing intra‐ or postoperative complications
Rojas et al. [43]	2021	52	30°	Neutral	30 patients ACLR alone vs. 22 ACLR + LET. Effect of LET on the MRI maturity signal of ACL graft	ACLR with LET exhibited at the 10‐month follow‐up different MRI signal intensities compared to isolated ACLR, suggesting LET may affect the graft's ligamentization process.
Rowan et al. [44]	2019	171	30°	Neutral	125 patients ACLR group and 46 patients ACLR + LET group with a median follow‐up of 52 months and 27 months, respectively	ACLR + LET results in better patient‐reported outcomes, quicker return to sport, and lower re‐injury rates
Alm et al. [2]	2020	75	45°	Not specified	14 patients received an isolated revision ACLR vs. 59 patients revision ACLR + LET	LET significantly reduces failure rates and the incidence of positive pivot shifts and enhances postoperative functional scores
Eggeling et al. [12]	2021	78	45°	Not specified	Revision ACLR vs. revision ACLR + LET in patients with pre‐op low‐grade anterior knee laxity	LET did not significantly reduce failure rates or improve clinical outcomes compared to patients who only underwent revision ACL reconstruction in patients with low‐grade anterior knee laxity.
Behrendt et al. [3]	2023	52	45°	Neutral	Onlay anchor fixation vs. transosseous fixation for LET during revision ACLR	No significant differences in patient‐reported outcomes, clinical examination, or instrumented testing between the two methods.
Chiba et al. [6]	2021	18	60°	Neutral	Impact of LET in combination with ACLR on knee kinematics during downhill running.	At 6 months, LET with ACLR significantly reduced anterior tibial translation at foot strike compared to ACLR alone, but this effect was not observed at 12 months. Additionally, LET with ACLR did not significantly affect tibial rotation at either 6 or 12 months post‐surgery.
Gibbs et al. [19]	2022	20	60°	Neutral	Patients were randomised to undergo anatomic ACLR with or without LET	Quantitative pivot shift does not correlate with postoperative kinematics following ACLR with or without LET.
Sheean et al. [45]	2020	20	60°	Neutral	Determine if augmenting ACLR with LET would significantly affect rotatory knee laxity	Utilising iQPS testing, the study found that both ACLR alone and ACLR with LET resulted in significant decreases in rotatory knee laxity. The addition of LET did not lead to overconstraint of knee kinematics compared to the contralateral knee.
Getgood et al. [18]	2020	618	60°–70°	Neutral	Compared ACLR + LET vs. ACLR alone. From the STABILITY Study.	LET significantly reduces the risk of ACLR failure, particularly graft rupture and persistent rotatory laxity, in young active individuals at high risk of failure.
Di Benedetto et al. [11]	2021	16	90°	Maximum external rotation	Kinematic and kinetic analysis of ACLR associated with LET in revision surgeries	patients regained pre‐injury sagittal knee stability and gait dynamics one year after surgery. The combination of these procedures can effectively restore the kinematics and stability of the knee joint
Joseph et al. [29]	2020	87	90°	Neutral	ACLR stand‐alone in 52 knees and with a Lemaire procedure in 35 knees	Lemaire procedure does not compromise isokinetic muscle recovery at the time of return‐to‐play, offering equivalent outcomes to ACLR alone.
Foissey et al. [13]	2022	39	0°	Not specified	ACLR + LET in skeletally immature patients	Low failure rate and minimal growth disturbances over an average follow‐up of 57 months, with a high rate of return to sports

Abbreviations: ACL, anterior cruciate ligament; ACLR, anterior cruciate ligament reconstruction; ALLR, anterolateral ligament reconstruction; BTB, bone‐patellar tendon‐bone; IKDC, International Knee Documentation Committee; LET, lateral extra‐articular tenodesis; MRI magnetic resonance imaging; SB ACLR + LET, single‐bundle anterior cruciate ligament reconstruction combined with lateral extra‐articular tenodesis.

**Table 3:** The literature search exclusively identified cadaveric study results.

Authors	Date of pub.	N. of patients	Degree of flexion	Tibial rotation	Methods	Considerations
Herbst et al. [22]	2017	7	30°	Not specified	Cadaveric study: ACLR + LET in Seven human lower limb cadavers	LET combined with ACLR reduced anterior tibial translation and internal tibial rotation in knees with both ACL and anterolateral capsule injuries. LET with ACLR can overconstrains the knee
Neri et al. [38]	2021	4	30°	Neutral	Cadaveric study: biomechanical analysis to compare lateral tibiofemoral contact pressures across different anterolateral procedures + ACLR	Procedures like Lemaire, and modified MacIntosh significantly increased LTF contact pressures, particularly in internal rotation. In contrast, ALLR and modified Ellison procedure did not significantly alter overall LTF contact pressures
Novaretti et al. [39]	2020	9	30°	Not specified	Cadaveric study: effects of LET on ACL graft in situ forces and tibiofemoral contact pressures	LET does not significantly overconstrain the knee nor increase lateral compartment pressures, and it effectively reduces in situ ACL force in the setting of anterolateral capsule injury
Lagae et al. [38]	2020	12	60°	Neutral	Cadaveric study: evaluates the addition of a monoloop LET to ACLR	mLET combined with ACLR would better restore knee laxity than ACL reconstruction alone in cases with combined anterior and rotational laxity.
Marom et al. [35]	2020	7	60°	Neutral	Cadaveric study: demonstrates the effect of LET + ACLR	LET + ACLR effectively reduces the force on the ACL graft and anterior tibial translation during pivoting and anterior drawer load applications and can offload the ACL graft, potentially protecting it post‐surgery
Marom et al. [34]	2021	7	60°	Neutral	Cadaveric study: explored how LET affects lateral compartment contact mechanics under pivoting manoeuvres	Augmenting ACLR with LET shifts the center of contact stress anteriorly on the lateral tibial plateau increasing anterior contact force. This can potentially impact post‐operative outcomes and the health of the lateral compartment
Mayr et al. [36]	2022	6	60°	Neutral	Cadaveric study: measure graft forces of ACLR and LET	Found that LET significantly reduces ACLR graft forces during internal tibial torque loading and could protect the ACLR graft, especially during rotational loadings and decrease rupture rates
Pearce et al. [41]	2023	12	60°	Neutral	Cadaveric study: explored the effects of LET on ACL graft forces and tibial motion at different tibial slopes	LET reduced graft force at certain knee flexion angles and tibial slopes, suggesting that LET could provide additional stability in ACL reconstructions, particularly at critical angles
Sigloch et al. [48]	2022	7	60°	Neutral	Cadaveric study: focused on the biomechanical impact of femoral insertion point variability in LET during active knee flexion‐extension cycles	small‐scale deviations in the LET insertion point during surgery do not significantly impact the efficacy of LET in supporting ACLR, providing valuable insights for clinical practice
Delaloye et al. [9]	2020	6	70°	Neutral	Cadaveric study: effect of ALLR and LET on knee stability following ACLR tested with a robotic system	Both ALLR and LET, when combined with ACLR, restore knee stability to levels similar to an intact knee, without significant differences between the two techniques in terms of anterior translation and internal rotation, without overconstraining the knee
Geeslin et al. [16]	2017	10	70°	Not specified	Cadaveric study: robotic Study Comparing ALL Reconstruction and LET fixation	Both procedures could significantly reduce tibial internal rotation, suggesting they may effectively address rotational laxity in ACL reconstructions.
Ozbek et al. [40]	2023	10	70°	Neutral	Cadaveric study: biomechanical effects of LET in ACL‐reconstructed knees with partial medial meniscectomy	LET significantly decreased anterior tibial translation and internal rotation, particularly at lower knee flexion angles, suggesting that LET could effectively reduce knee laxity in these complex scenarios
Shimakawa et al. [47]	2019	8	70°	Neutral	Cadaveric study: focused on lateral compartment contact pressures increasing after LET and subsequent subtotal meniscectomy	LET when performed with ACLR, does not significantly alter these pressures, even in the presence of lateral meniscal injury. This provides reassurance regarding the safety of LET in ACLR without risking increased lateral compartment pressures
Jette et al. [28]	2019	12	0°	Neutral	Cadaveric study: Compares the biomechanical outcomes ALL reconstruction with LET alongside ACLR	Both procedures effectively restored internal rotation to near intact‐knee levels at lower flexion angles without significant differences between them, although some overconstraint was observed at higher flexion angles
Inderhaug et al. [25]	2017	12	0°/30°/60°	Neutral	Cadaveric study: Effect of knee flexion angle at graft fixation on tibiofemoral joint kinematics during ACLR combined with LET or ALL reconstruction	LET procedure could restore knee kinematics across all flexion angles of graft fixation, while the ALL procedure only restored kinematics when the graft was fixed in full extension

Abbreviations: ACL anterior cruciate; ACLR, anterior cruciate ligament reconstruction; ALL, anterolateral ligament; ALLR, anterolateral ligament reconstruction; LET, lateral extra‐articular tenodesis; LTF, lateral tibiofemoral.

One study reported graft fixation in full extension [13] in paediatric patients at a high risk of graft rupture.

One study reported graft fixation at 20° of flexion [15], combining LET and a hamstring ACL reconstruction.

Ten out of the 21 studies [1, 4, 5, 8, 10, 20, 21, 42, 43, 44] reported graft fixation at 30°, all of which were in primary ACLs, comparing hamstrings [1, 8, 10, 15, 21, 42, 43, 44], bone‐to‐bone‐patellar tendon graft [4, 5] and quadriceps tendon [20].

Three studies [2, 3, 12] describe the fixation of the graft al 45° of flexion, again, all on revision ACLR.

Four studies [6, 18, 19, 45] described graft fixation with the knee flexed at 60°, including the Stability 2 study. Finally, two studies [11, 29] reported results of fixation at 90°. The main findings of the selected studies are reported in Table 2. The knee rotational degrees during the fixation were found to be in the majority of the cases neutral and are reported in the Table 2.

## DISCUSSION

The primary finding of this manuscript is the significant variability in the literature concerning the optimal knee flexion angle for graft fixation in the Lemaire procedure. This variation appears to be influenced by surgeon experience and individual practice preferences.

This systematic review aimed to elucidate the reported angles for graft fixation in a LET procedure performed concomitantly with ACL reconstruction, as reported in clinical studies with a minimum 12‐month follow‐up. The findings of the review reveal a wide range of fixation angles, from full extension to 90° of flexion, all reportedly yielding good clinical outcomes for what appears to be the same procedure. This variability underscores the need for standardised protocols to ensure consistency and optimise patient outcomes. The LET procedure aims to prevent antero‐lateral instability and neutralise pivot shift, so this aspect should be taken in consideration when fixing the graft. This pivoting effect occurs in a range between 15° and 35°, with an average 20.7° degrees of flexion reported Kitamura et al. [30]. This value seems to be confirmed by Hoshino et al., that reported a peak of anterior tibial translation at 22.0° during the pivot shift manoeuvre [24]. As evidenced in the present study, in real surgical practice, many different angles of fixation for the Lemaire procedure are routinely used in anterior cruciate ligament reconstructions. However, most of the surgeons try to fix the graft at 30°, close to the average degrees of maximum antero posterior translation in pivot shift manoeuvre.

When performing an ACL reconstruction, the aim is to re‐establish the native ACL function, providing stability and allowing full range of motion, but also establishing the screw‐home mechanism of the knee [26]. This is achieved by fixing the ACL‐graft at a maximum of 30° [7, 33]. The single study fixing the graft at full extension was performed in paediatric patients with the results reported at an average follow‐up of 57 months [25]. The study finds that ACLR + LET procedures offer promising outcomes for growing children, with low complication rates including graft rupture and growth disturbance. The authors prefer to fix the LET in full extension to ensure optimal graft tension and knee stability throughout the entire range of motion, particularly during activities requiring full knee extension.

The single study fixing the graft at 20° of flexion did not demonstrate significant benefits in terms of sagittal knee stability [14]. The authors suggest that this angle might not be optimal for enhancing the procedure's efficacy.

In contrast, the almost half of the studies in the present systematic review performed graft fixation at 30°. All authors report improved knee stability and clinical outcomes, including lower pivot shift incidence, better IKDC scores, and a quicker return to sport. These findings are particularly relevant for athletes and individuals at high risk of rotational instability or re‐injury, underscoring the significance of selecting an appropriate flexion angle to maximise the procedure's benefits. Among these, the most significant is certainly the study by Ahn et al. [1] that compares single‐bundle anterior cruciate ligament reconstruction combined with lateral extra‐articular tenodesis (SB ACLR + LET) against double‐bundle ACL reconstruction (DB ACLR).

Rowan et al. [44] in a long‐term follow‐up study on 171 patients demonstrated that ACL reconstruction combined with LET results in better patient‐reported outcomes, quicker return to sport, and lower re‐injury rates compared to ACL reconstruction alone. Both the two previous studies fix the LET at 30° of knee flexion to ensure the graft is appropriately tensioned, which helps to enhance rotational stability and reduce the risk of over‐constraining the knee joint, thereby optimising the overall effectiveness and safety of the procedure.

On the other hand, Castoldi et al. [5] conducted a 19‐year follow‐up study on 120 patients who underwent ACLR with bone‐patellar tendon‐bone graft, with and without LET. The authors prefer to fix the graft in neutral rotation to ensure proper tensioning, which enhances rotational stability and prevents excessive tightening of the lateral knee compartment.

Green et al. [20] and Perelli et al. [42] described this surgical technique in adolescent patients, reporting respectively favourable clinical outcomes over a 2‐year follow‐up, including a 0% graft rupture rate and a 100% return‐to‐sports rate. Green et al. prefer to use anchor fixation for the graft while Perelli et al. bioabsorbable screws. Both the authors fix the LET at 30° of knee flexion in neutral rotation to ensure the graft is tensioned properly, enhancing rotational stability and preventing over‐tightening of the knee join

The studies examining graft fixation at higher angles, such as 45° and 60°, present mixed results. While some research suggests that these angles can reduce failure rates and enhance functional scores, especially in revision ACLR, others indicate potential risks, including over‐constraint of the knee and increased lateral compartment pressures. These discrepancies highlight the need for careful patient selection and personalised surgical planning to balance stability improvements with the risk of long‐term complications.

The research by Alm et al. [2] indicates that LET significantly reduces failure rates and the incidence of positive pivot shifts and enhances postoperative functional scores in such patients. Eggeling et al. [12] explored the effects of LET in revision ACL reconstruction for patients with low‐grade anterior knee laxity. They found that adding LET did not significantly reduce failure rates or improve clinical outcomes compared to patients who only underwent revision ACL reconstruction. They use an interference screw for the fixation at the femoral site and they prefer the 45° of knee flexion to ensure optimal tension and rotational stability while preventing over‐constraint of the knee joint.

The exploration of graft fixation at extreme angles, such as full extension or 90° of flexion, presents novel considerations. While fixation in full extension may restore knee kinematics effectively, the potential for overconstraint at higher flexion angles warrants caution. Similarly, the promising outcomes associated with fixation at 90°, including improved stability and return‐to‐sport rates, suggest that this angle may offer a viable option for specific patient populations, particularly those with severe rotational instability. The pilot study by Di Benedetto et al. [11] found that patients who underwent this combination treatment regained pre‐injury sagittal knee stability and gait dynamics one year after surgery. Joseph et al. [29] investigates the impact of adding a modified Lemaire procedure to ACLR in patients with severe rotational knee instability. The 90° angle and neutral position were selected to ensure the proper tensioning of the iliotibial (IT) band graft with an interference screw, aiming to reduce anterolateral rotatory instability effectively. Fixing the LET in this position helps to recreate the natural biomechanics of the knee by stabilising the anterolateral aspect and thereby improving the rotational stability provided by the ACL reconstruction

The relationship between knee flexion angle during graft fixation in the Lemaire procedure and postoperative outcomes is nuanced. While there is a trend towards fixation at 30° of flexion for optimising knee stability and function, the variability in individual anatomy, injury characteristics, and surgical techniques necessitates a personalised approach. Future research should focus on longitudinal studies with larger sample sizes to further refine the optimal knee flexion angle for graft fixation, considering the dynamic interactions between knee biomechanics, surgical technique, and patient‐specific factors. Additionally, the development of more sophisticated biomechanical models may aid in preoperative planning and simulation, ultimately improving surgical precision and patient outcomes in ACL reconstruction combined with lateral extra‐articular tenodesis.

The study's limitations include heterogeneity in study designs, surgical techniques, and fixation angles, making direct comparisons challenging. Short to medium‐term follow‐ups restrict insights into long‐term outcomes like graft durability and osteoarthritis.

## CONCLUSION

The systematic review indicates that LET, when combined with ACLR, is effective in restoring knee stability across various flexion angles. Although no consensus exists on the optimal knee flexion angle for graft fixation due to differences in surgical techniques and some patients' specific factors, most authors report flexion angle around 30°, which seems to be the main research focus as well, potentially suggesting a good balance between stability and mobility with reduced risks of overconstraint and degenerative changes. Further clinical and biomechanical studies with specific focus on flexion angle of LET during ACLR are needed.

## AUTHOR CONTRIBUTIONS

Riccardo Compagnoni design of the work. Francesco Puglia interpretation of data and have drafted the work. Antonio Klasan and Alberto Grassi substantial contributions to the conception. Stefano Zaffagnini the acquisition of data. Francesco Puglia interpretation of data. Pietro Simone Randelli revised the work. Jacques Menetrey design and revised the work. All authors read and approved the final manuscript.

## CONFLICT OF INTEREST STATEMENT

The authors declare no conflicts of interest.

## ETHICS STATEMENT

This research did not involve human participants or animals, and therefore ethical approval was not required. The data analyzed were publicly available and anonymized.

## Data Availability

The data that support the findings of this study are available from the corresponding author upon reasonable request.
